# Mechanical Recanalization following i.v. Thrombolysis: A Retrospective Analysis regarding Secondary Hemorrhagic Infarctions and Parenchymal Hematomas

**DOI:** 10.1155/2015/159815

**Published:** 2015-11-10

**Authors:** J. Höltje, F. Bonk, A. Anstadt, C. Terborg, C. Pohlmann, P. P. Urban, R. Brüning

**Affiliations:** ^1^Institute of Radiology and Neuroradiology, Asklepios Hospital Barmbek, Hamburg, Germany; ^2^Department of Neurology, Asklepios Hospital St. Georg, Hamburg, Germany; ^3^Department of Neurology, Asklepios Hospital Barmbek, Hamburg, Germany

## Abstract

*Introduction*. In acute stroke by occlusion of the proximal medial cerebral artery (MCA) or the distal internal carotid artery, intravenous thrombolysis is an established treatment. Another option is mechanical recanalization. It remains unclear if the combination of both methods poses an additional bleeding risk. The aim of this retrospective analysis is to determine the proportion of hemorrhagic infarctions and parenchymal hematomas. *Methods*. Inclusion criteria were an occlusion of the carotid T or proximal MCA treated with full dose thrombolysis and mechanical recanalization. 31 patients were selected. Devices used were Trevo, Penumbra Aspiration system, Penumbra 3D Retriever, and Revive. The initial control by computed tomography was carried out with a mean delay to intervention of 10.9 hours (SD: 8.5 hours). *Results*. A slight hemorrhagic infarction (HI1) was observed in 2/31 patients, and a more severe HI2 occurred in two cases. A smaller parenchymal hematoma (PH1) was not seen and a space-occupying PH2 was seen in 2/31 cases. There was no significant difference in the probability of intracranial bleeding after successful (thrombolysis in cerebral infarctions 2b and 3) or unsuccessful recanalization. *Conclusion*. The proportion of intracranial bleeding using mechanical recanalization following intravenous thrombolysis appears comparable with reports using thrombolysis alone.

## 1. Introduction

Various randomized trials of intravenous thrombolytic therapy, alone and in combination with subsequent mechanical recanalization in acute ischemic stroke, have been conducted. As side effects of this regime, either secondary hemorrhagic infarctions (HI) or parenchymal hematomas (PH) after different types of thrombolytic therapy have frequently been reported, but they also occur as natural events in the evolution of a cerebral infarct without [1, 2] or with intravenous thrombolysis [3]. Large trials on the use of tissue plasminogen activator (rtPA) provided evidence of a benefit to ischemic stroke patients, despite hemorrhagic events also occurring here [4, 5].

Since the introduction of devices to retrieve a thrombus directly from the artery by mechanical means, catheter-based methods have been integrated into various treatment algorithms [6, 7], with and without thrombolytic agents. In most centers, in acute stroke due to the occlusion of the proximal medial cerebral artery (MCA) or the distal carotid artery (ICA), intravenous thrombolysis using rtPA will be carried out, and if symptoms or signs of continuing vessel occlusion persist this will be followed by mechanical recanalization.

However, it remains unclear whether the combination of rtPA and the recent use of mechanical recanalization devices outside of safety studies carries an increased risk of either secondary hemorrhagic infarction (HI) or parenchymal hematoma (PH). The aim of this retrospective single center analysis was to determine whether the proportion or severity of these secondary hemorrhagic infarctions or parenchymal hematomas, as defined by Berger and coworkers [8], which were also used in the European Cooperative Acute Stroke Study (ECASS analysis), increased in this therapeutic setting.

## 2. Methods

All patients were diagnosed and treated based on the established clinical standards in our center. All patients, or their next of kin, gave informed consent to a retrospective analysis. This retrospective search of our database was conducted over 24 months.

Inclusion criteria consisted of a body-weight-adjusted full dose intravenous rtPA treatment (Actilyse, (alteplase) Boehringer Ingelheim Pharma, Germany) in acute stroke patients.

Additional inclusion criteria consisted of occlusion of the anterior circulation (thrombolysis in cerebral infarction TICI = 0), as documented by multimodal CT including unenhanced imaging and CT-angiography in these cases and mechanical recanalization being performed. A control CCT had also to be available.

Exclusion criteria consisted of age under 18 years, pregnant females, National Institute of Health Stroke Scale (NIHSS) < 5, acute accident with head trauma, or absence of intravenous thrombolysis if there were contraindications like phenprocoumon (2 cases), substitution with prothrombin complex concentrate (PPSB), rivaroxaban (1 case), thrombolysis in the last four weeks (1 case), coincidental accidents (2 cases), thrombosis of the basilar artery or stenting of the proximal carotid internal artery and the previous application of tirofiban (11 cases), and the finding of an intracranial aneurysm (1 case). Patients with early signs of large infarctions on the admission CT greater than one-third of the middle cerebral artery (MCA) region, severe edema, or previous intracranial hematoma (ICH) were also excluded. Patients lost to follow-up were similarly excluded.

In total, we treated 48 patients matching the inclusion criteria full dose thrombolysis with thrombectomy, and we, for the study, excluded a total of 17 (35.4%) patients due to the exclusion criteria above.

31 patients matched the inclusion and exclusion criteria and were analyzed further. There were 13 male and 18 female patients, with the mean age being 70.52 years (standard deviation 10.43 years, ranging from 47 to 87 years).

Mechanical recanalization was performed under general anesthesia in all patients. Based on a transfemoral approach with 6-7F introducer sheets, 6 F guiding catheters (usually ENVOY Guiding Catheter Codman, Inc., Raynham, MA 02767, USA) and various microwire retriever sets with a European CE certificate were used: Trevo and Trevo pro *n* = 18 (Stryker USA), Penumbra Aspiration System *n* = 10, Penumbra 3D *n* = 2 (Penumbra Inc., USA), and Revive *n* = 1 (Codman Endovascular, USA). For the determination of revascularization success, we used the TICI score [9, 10], a modified score of the Thrombolysis In Myocardial Infarction (TIMI) flow classification [11]. We used the terminology and definition of the TICI score like reported in the IMS II trial [9]. TICI 0 means no perfusion, TICI 1 means a perfusion past the initial occlusion but with limited filling of distal branch with little or slow perfusion, and TICI 2a means a perfusion of less than half of the vascular distribution of the occluded artery, for example, only filling of one M2 division. TICI 2b describes a perfusion of equal or more than half of the vascular distribution of the occluded vessel, for example, reperfusion of two or more M2 divisions. TICI 3 means a full perfusion with no persisting occlusions. In IMS II, a good clinical outcome modified Rankin Scale (mRS) 0–2 correlated with a recanalization of TICI 2b or 3 [9]. The modified Rankin Scale rates the handicap after stroke (0 = no symptoms; 5 = severe disability) [12].

Controls were performed by unenhanced CCT (Brilliance 40 Philips Medical Systems, Netherlands, or Optima 660, GE Health Care, USA), using sequential 1.25 and 5 mm slices in the supratentorial region of interest. Unless otherwise stated, the first available control was used in this analysis, mean interval from mechanical recanalization was 10.9 hours (standard deviation: 8.5 hours). All control-CT were retrospectively reviewed by two staff neuroradiologists regarding the presence or absence of bleeding and were grouped according to a rating score published previously by Berger and coworkers [8], which has been used in international trials such as the Penumbra Pivotal Stroke Trial and the European Cooperative Acute Stroke Study (ECASS) [6, 13]:

1. hemorrhage infarction Type 1 (HI1): small petechiae along the margins of the infarct;
2. hemorrhage infarction Type 2 (HI2): more confluent petechiae within the infarcted area but without a space-occupying effect;
3. parenchymal hematoma Type 1 (PH1): a hematoma in less than 30% of the infarcted area with some space-occupying effect;
4. parenchymal hematoma Type 2 (PH2): a hematoma in more than 30% of the infarcted area with a substantial space-occupying effect or as any hemorrhagic lesion outside of the infarcted area.

Statistical analysis to correlate the success of recanalization with the type and percentage of bleeding was performed on an SAS analysis system using the Fisher Exact Test.

## 3. Results

As determined by the inclusion criteria, only patients with intravenous thrombolysis and following mechanical recanalization were analyzed.

In our retrospective analysis, 54.8% recanalization of the parent vessel to a TICI 2b or TICI 3 was reached, and in 71% any successful recanalization was achieved (TICI 2 and TICI 3) (including 2a *n* = 5/16.1%).

**Figure 1:**
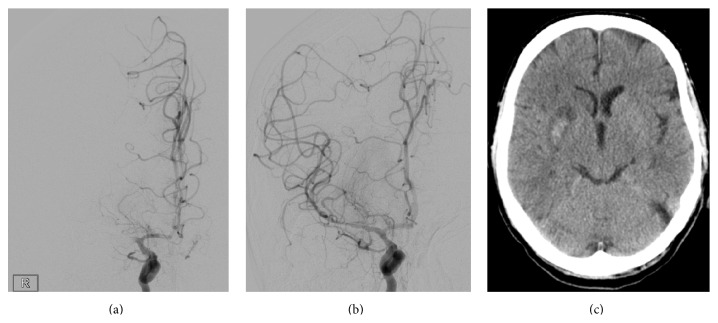
(a) Occlusion of middle cerebral artery TICI 0; (b) DSA following mechanical recanalization TICI 3; (c) unenhanced CCT on day 2 (24 hours) with a slight hemorrhagic infarction HI1.

A discrete hemorrhagic infarction, HI1, as shown in Figure 1(c), was detected in 2/31 (6.5%), and a more prominent hemorrhagic infarction, HI2, was found in two cases (6.5%). A subtle parenchymal hematoma, PH1, was not found and a more prominent and space-occupying parenchymal hematoma, PH2, was detected in 2/31 cases (6.5%). For details, refer to Table 1.

There were no bleeding cases in our cohort in the absence of recanalization, but there were bleeding events even if successful mechanical thrombectomy was incomplete. If the patients were subgrouped (TICI 0-2a and TICI 2b-3), the probability of secondary hemorrhagic infarctions (HI) and parenchymal hematomas (PH) exhibited a trend, but there was no statistically significant relationship with the success of recanalization (bleeding cases were observed in the cohort TICI 0-2a: 2/31 HI, 1/31 PH; in the cohort TICI 2b and TICI 3: 2/31 HI, 1/31 PH2) Fischer's Exact Test two-sided PR ≤ *p* 1.00. In the nine cases without recanalization (TICI 0-1), there was no bleeding; neither there was hemorrhagic infarction nor parenchymal hematoma. We used different recanalization devices. Most times *n* = 18; we used a Trevo/Trevo pro. We noticed six hematomas with this system and one with the Penumbra Aspiration system.

## 4. Discussion

In our retrospective analysis of a single center series, we found 6.5% of parenchymal and symptomatic bleeding cases and a total bleeding rate of 19.4% in patients treated with different mechanical recanalization devices after a full dose of intravenous thrombolysis.

Regarding the overall rate of cerebral bleeding following therapy, publications using equivalent mechanical devices, such as the Penumbra Pivotal Stroke Trial, reported a 24-hour intracranial hematoma (ICH) rate of 28% [6]. The hemorrhagic rate in our series was lower compared to trials such as the Second Prolyse in Acute Cerebral Thromboembolism Trial (PROACT II) (35.9%) [14], or the Mechanical Embolus Removal in Cerebral Ischemia (MERCI) Phase II trial, reporting a rate of 35.5% [15], and to the Multi-MERCI trial reporting a rate of 38.7%. In Table 2, we compared our results with ECASS II (i.v. thrombolysis alone), the MERCI trial, MR CLEAN, and some smaller trials due to the risk of bleeding. There is no higher risk of hematoma in the interventional trials. However, some previous trials in acute stroke had different definitions for the term symptomatic hematoma or symptomatic hemorrhagic infarction. Another limitation of this retrospective analysis was the different intervals between intervention and cerebral imaging and other variables such as postinterventional blood pressure management to prevent recanalization trauma in the present series and previous trials.

According to Berger et al. [8], we used the definition “symptomatic” bleeding for bleeding which is equal to a more severe parenchymal hematoma PH2. We did not consider PH1 bleeding, as there was none in our cohort. Yilmaz et al. [16] reported no significant influence of peri-interventional SAH due to mechanical thrombectomy on the neurologic outcome, but there was a higher rate of ICH in the first 24 h if peri-interventional SAH occurred. Regarding symptomatic bleeding only, a rate of 6.5% in our retrospective analysis is comparable with the reported data in the PROACT II (10.9%) [14] and Multi-MERCI (9.8%) trials [7], the most recent trials from 2014 and 2015 (MR CLEAN, SWIFT PRIME, ESCAPE, and EXTEND IA) [17–20] using almost exclusively stent retriever refer about 3.6 to 11% parenchymal hematoma in the interventional arm as compared to 2.7 to 7% in the arm of the systemic thrombolysis alone. Moreover, the Interventional Management of Stroke trial (IMS 3) reported a symptomatic hemorrhagic rate at 30 hours of about 6% in both groups (one receiving rTPA and mechanical recanalization and one receiving rTPA alone), and a nonsignificant difference with the group treated by rTPA alone (*p* = 0.83) [21], although based on different catheter technologies such as low-energy ultrasound or the MERCI retriever system [22]. In our analysis, most of the hematomas occur when we used a Trevo/Trevo pro device and only one PH2 with a microwire retriever. In comparison to the newer studies using stent retrievers in majority or exclusively this finding in our cohort is probably a bias due to our small sample size.

In our analysis, we grouped the patients with successful recanalization (TICI 2b and TICI 3) and those with none or only minor recanalization of the entire vascular territory (TICI 2a) because we assumed that mechanical recanalization was not dangerous, even following full dose thrombolysis, if we have normal postrecanalization perfusion pressure. Nelles et al. [23] showed ICH of 5% in TICI 2b and TICI 3, but only 27.6% of their patients received half dosage i.v. thrombolysis and the other 62.4% received none.

If the patients with no efficient recanalisation were analysed with respect to hematoma (TICI 0 and TICI 1), then there is no bleeding.

The use of the REVIVE stent retriever system was published by the Heidelberg group with good results concerning recanalization, but with a reported rate of symptomatic intracranial bleeding of 20% [24].

Yilmaz et al. [16] reported that, in a large series, there was no significant influence of peri-interventional SAH due to mechanical thrombectomy on the neurologic outcome, but a higher rate of ICH in the first 24 h if peri-interventional SAH occurred.

Fiorelli et al. showed on the basis of the ECASS I data, that, in both the placebo and rtPA groups, only parenchymal hematomas (PH) in >30% of the infarcted area, with a significant space-occupying effect, increased the risk of early neurological deterioration and of 3-month death [25].

Based on the ECASS II data, the same group concluded that only large PH greater than 30% of the infarcted area with a space-occupying effect (PH2) independently modified the risk of a worsened clinical outcome, both early and late after stroke onset (odds ratio 20). Smaller, but still homogeneous, PH (PH1) increased the risk of early deterioration, but not that of a worsened long-term outcome [8].

In order to optimize our workflow, we used catheter techniques that, as far as possible, were adapted to given standards to minimize the risk to patients.

Regarding the rate relationship, as measured by the TICI score, it was not the aim of this analysis to investigate recanalization rates, and interpretation may have been affected by negative selection in the patient cohort that was investigated. Nevertheless, the rate of recanalization found in our retrospective review was well within the previously published values of other renown centers [19–23]. The recanalization rate of TICI 2b and TICI 3 is 54.8% compared to the rate in the MR CLEAN [20] study 58.7%, which may be because we also used different kind of stent retrievers and microwire retrievers.

Limitations of this study are acknowledged; they are based on its retrospective nature and the single center basis with a relatively small sample size.

In conclusion, treatment of stroke using currently available retriever and especially stent retriever devices for mechanical recanalization following full dose intravenous thrombolysis does not increase the risk of hemorrhagic infarctions and parenchymal hematomas as compared to thrombolysis alone and is judged as reasonably safe.

## Figures and Tables

**Table 1:** Patient data and HI and PH in comparison to published values.

Age	Set	TICI posttreatment	Hours to CT	HI	PH
80	Penumbra	0	23	0	0
71	Penumbra	0	25	0	0
87	Penumbra	0	15	0	0
63	Penumbra	0	6	0	0
73	Penumbra 3D	0	3	0	0
74	Trevo	0	15	0	0
71	Trevo	0	14	0	0
49	Trevo	0	10	0	0
79	Trevo	0	0	0	0
82	Penumbra	2a	14	0	0
81	Penumbra	2a	20	0	0
59	Penumbra	2b	21	0	0
71	Penumbra	2b	14	0	0
83	Revive	2b	3.5	0	0
74	Trevo	2b	10	0	0
49	Trevo	2b	19	0	0
53	Penumbra	2b	22	0	0
68	Penumbra 3D	3	5	0	0
77	Trevo	3	13	0	0
75	Trevo	3	16	0	0
75	Trevo	3	24	0	0
58	Trevo	3	0.3	0	0
76	Trevo	3	0	0	0
69	Trevo	3	0	0	0
80	Trevo	3	23	0	0
74	Trevo	2a	15	1	0
62	Trevo	2a	0	1	0
74	Trevo	2b	1	2	0
47	Trevo	2b	1	2	0
78	Penumbra	2a	1	0	2
74	Trevo	3	5	0	2

**Table 2:** Comparison to other studies.

	Present study	Hacke et al. [13]	Smith et al. [15]	Smith et al. [7]	Penumbra Pivotal Stroke Trial [6]	Nelles et al. [23]	MR CLEAN [20]
*n* (100%)	31	406	141	164	125	65	500
Target vessel	ICA, MCA	ICA, MCA	ICA, MCA, VA, BA	ICA, MCA, VA, BA	ICA, MCA, VA, BA	ICA, MCA	ICA, MCA, ACA
i.v. thrombolysis with rtPA	31 (100%)	406 (100%)	No	48 (29%)^*∗*^	n/a^#^	n/a	445 (89%)
Mechanical recanalization	31 (100%)	no	141 (100%)	164 (100%)	125 (100%)	65 (100%)^##^	195 (39%)
Hematoma	6 (19.4%)	120 (29.5%)	50 (35.5%)	16 (9.8%)	35 (28%)	3 (5%)^##^	35 (7%)

^*∗*^i.v. and also i.a. thrombolysis.

^#^Only the percentage of i.v. rtPA in case of bleeding, not the overall i.v. rtPA rate.

^##^Only TICI 2b and TICI 3, where they administered only 50% of the usual dose.
